# Establishment of four head and neck squamous cell carcinoma cell lines: importance of reference DNA for accurate genomic characterisation

**DOI:** 10.1017/S0022215122000846

**Published:** 2023-03

**Authors:** K B Patel, S Prokopec, J W Barrett, J S Mymryk, P C Boutros, A C Nichols

**Affiliations:** 1 Department of Head and Neck – Endocrine Oncology, Moffit Cancer Center, Tampa, Florida, USA; 2 Informatics and Biocomputing Platform Ontario Institute for Cancer Research, Toronto, Canada; 3 Department of Human Genetics, University of California, Los Angeles, USA; 4 Institute for Precision Health, University of California, Los Angeles, USA; 5 Department of Otolaryngology – Head and Neck Surgery, Western University, London, Ontario, Canada; 6 Department of Oncology, University of Western Ontario, London, Canada; 7 Department of Microbiology and Immunology, University of Western Ontario, London, Canada; 8 Department of Urology, University of California, Los Angeles, USA; 9 Eli and Edythe Broad Center of Regenerative Medicine and Stem Cell Research, University of California, Los Angeles, USA; 10 Jonsson Comprehensive Cancer Center, University of California, Los Angeles, USA

**Keywords:** Mutation, Cell Line, Head And Neck Squamous Cell Carcinoma, Human Papillomavirus, HPV-Positive Cell Lines, HPV

## Abstract

**Objective:**

There is significant interest in developing early passage cell lines with matched normal reference DNA to facilitate a precision medicine approach in assessing drug response. This study aimed to establish early passage cell lines, and perform whole exome sequencing and short tandem repeat profiling on matched normal reference DNA, primary tumour and corresponding cell lines.

**Methods:**

A cell culture based, *in vitro* study was conducted of patients with primary human papillomavirus positive and human papillomavirus negative tumours.

**Results:**

Four early passage cell lines were established. Two cell lines were human papillomavirus positive, confirmed by sequencing and p16 immunoblotting. Short tandem repeat profiling confirmed that all cell lines were established from their index tumours. Whole exome sequencing revealed that the matched normal reference DNA was critical for accurate mutational analysis: a high rate of false positive mutation calls were excluded (87.6 per cent).

**Conclusion:**

Early passage cell lines were successfully established. Patient-matched reference DNA is important for accurate cell line mutational calls.

## Introduction

Clinical trials are the ‘gold standard’ for establishing the efficacy of anti-cancer agents. However, only a limited number of hypotheses can be tested because of the high expense, the limited number of patients and the length of time necessary to complete these studies.^1^ The realisation that each cancer site consists of multiple molecular subtypes that may respond differently to a particular targeted agent further complicates trials,^2^ making companion biomarkers a high priority. Model systems are therefore increasingly necessary to test the molecular basis of drug activity, which cannot be explored directly in patients.

Cancer cell lines are the most widely used model systems in cancer research; however, they are imperfect, as they lack a three-dimensional environment, stromal interactions, tumour-microenvironmental features like hypoxia, and immune system effects.^3,4^ Cell lines do, however, present with several advantages, including the fact that they cost relatively little to maintain, can be easily manipulated in biological assays, are relatively amenable to genetic manipulation and provide rapid results. Indeed, they are the only model system that can be feasibly screened with a large number of agents to thoroughly explore mechanisms of drug sensitivity and resistance.^5^ In particular, there have been large-scale cell line drug screening efforts to correlate genetic profiles with drug response.^6–8^

Although there are well-established high-passage cell lines, patient-matched reference DNA is not available for accurate characterisation. Early passage cell lines are thought to better retain the molecular features of the primary tumours and have a stronger correlation with patient drug response.^9,10^ They have the potential additional advantage that patient-matched reference DNA and the primary tumour may be available to confirm that the cell line was indeed derived from the source patient and improved the accuracy of the genomic profiling.

For these reasons, we describe the establishment and characterisation of four head and neck cancer cell lines, including two human papillomavirus (HPV)-positive head and neck squamous cell carcinoma (SCC) cell lines. We confirmed the origin of all the cell lines through short tandem repeat profiling, and used whole exome sequencing of triplet samples (normal, primary tumour, cell line) to compare the genetic landscape of the cell line with the primary tumour and determined the importance of the normal reference DNA for making accurate mutational calls.

## Methods

### Patient recruitment and tumour processing

Patients with head and neck SCC undergoing biopsy or resection were consented for tumour collection with University of Western Ontario Health Sciences Research Ethics Board approval 16579E.

Ten millilitres of blood were drawn intra-operatively into heparinised tubes. The diagnosis of viable head and neck SCC was confirmed by frozen section in the operating theatre, and a portion of the remaining specimen was transported immediately in saline for processing. A portion of tumour was stored at −80°C. The remainder of the sample was further rinsed with saline and then minced into as small pieces as possible with a sterile razor blade. The tissue was incubated in 3 ml of Dulbecco's modified Eagle's medium/F12 medium supplemented with 10 per cent fetal bovine serum (Gibco®), penicillin (100 IU/ml) and streptomycin (100 μg/ml; Wisent, Quebec, Canada), amphotericin (0.25 mg/ml; Wisent) and collagenase (prepared to 1 mg/ml; Sigma®) for 1 hour at 37°C with regular agitation. Cells were washed twice with phosphate buffered saline, resuspended in primary tumour cell growth medium and divided into a six-well dish.

The medium was changed every 72 hours, and evaluated by microscopy for epithelial cell adherence and growth. If the cells were able to achieve confluence and clusters of cells appeared epithelial, passaging was attempted; if no yeast or fungus contamination was evident, the cells were transferred to cell line complete growth medium, which consisted of Dulbecco's modified Eagle's medium/F12 medium supplemented with 10 per cent fetal bovine serum and penicillin and streptomycin mixture. This was performed by incubation with 1 ml of trypsin (0.25 per cent, Wisent) for 2–5 minutes, followed by re-plating of 100 per cent of the cells into T75 flasks. This process was repeated for cell lines that continued to be able to achieve confluence and continued to have an epithelial appearance of greater than 50 per cent of cells. Representative stocks of each cell line were collected and frozen at −80°C during the passaging process in filter-sterilised freezing medium comprising 85 per cent fetal bovine serum and 15 per cent dimethyl sulfoxide.

### DNA extraction

DNA was extracted from cultured cells and matched primary tumours using the AllPrep DNA/RNA Kit (Qiagen, Toronto, Canada), following the instructions provided by the manufacturer. The DNA was extracted from the buffy coat layer separated from whole blood using the QIAamp DNA Blood Mini Kit (Qiagen). Minimum quality requirements for sequencing studies were 1 μg of genomic DNA with an optical density 260/280 ratio of between 1.7 and 1.9.

### Short tandem repeat profiling and virus detection

For each cell line that was developed, the corresponding matched blood and primary tumour were analysed by short tandem repeat profiling at The Centre for Applied Genomics (Toronto, Canada), as we have previously reported.^11^ The lines were genotyped with a panel of 10 selected markers. The cell line short tandem repeat profiling results were compared with the corresponding primary tumour tissue and blood to confirm the identity of the sample. Cell line DNA and RNA were also tested for the presence of all high-risk HPVs (types 16, 18, 31, 33, 35, 39, 45, 51, 52, 56, 58, 59, 68, 69 and 74) by real-time polymerase chain reaction, as we have previously described.^12^

### Immunoblotting

Whole cell lysates were prepared, immunoblotted and analysed as described previously.^13^ Membranes were exposed to chemiluminescence reagent (Luminata^™^ Crescendo or Luminata^™^ Forte (Millipore®)) using a Bio-Rad ChemiDoc^™^ MP Imaging System.

### Cell line morphology and doubling time

Photographs of all cell lines were taken at 4× and 10× magnification. In order to measure cell line doubling time, cells were counted and seeded into T25 flasks. Forty-eight hours later, the cells were collected and counted. Doubling time was calculated using an online calculator.^14^

### Matched cell line sequencing, tumours and reference blood

DNA samples were submitted to The Centre for Applied Genomics for whole exome sequencing. FASTQ files were aligned to GRCh38 using Burrows–Wheeler aligner algorithm with maximal exact matches (version 0.7.15). Duplicates were marked. Indel realignment and recalibration were performed using the Genome Analysis Toolkit (version 3.7.0). For tumours with a matched normal, germline single nucleotide polymorphisms were called using the Genome Analysis Toolkit HaplotypeCaller (version 3.7.0), and filtered for quality and read depth. The SomaticSniper (version 1.0.5.0) genome modelling tool was used to call single nucleotide variants in paired samples (tumour or cell line), with the reference being leukocyte DNA. A hypergeometric test was used to assess the probability of overlap between tumour and cell line samples, with *p* > 0.05 indicative of both samples having a greater overlap than expected by chance.

Germline single nucleotide polymorphisms were generated using the Genome Analysis Toolkit (version 3.7.0). HaplotypeCaller was then run on the realigned and recalibrated individual binary alignment maps, followed by genotyping, variant selection and hard filtering. Unpaired mutation calling was first performed using the MuTect identification algorithm (version 1.1.7), without supplying the paired normal for the tumour, and the cell line was realigned with recalibrated binary alignment maps for each patient. A panel of ‘normals’ was generated using MuTect (version 1.1.7) from 438 of Cancer Genome Atlas head and neck SCC normal exomes. The single nucleotide variants, unpaired with the panel of ‘normals’, were further filtered to retain only the ‘pass’ calls. The single nucleotide variants that were in the panel of ‘normals’ but were also in the ‘allowlist’ (because of being in the Catalogue of Somatic Mutations in Cancer (‘COSMIC’) database) were also removed. Single nucleotide variants in the capture regions and in chromosomes chr 1-22, X, Y and M were preserved. Somatic single nucleotide variants were filtered to remove non-functional (intronic or intergenic) variants prior to downstream analyses. Average true positive, false positive and false negative rates were then calculated for the four cell lines. Data visualisation employed the BPG (BoutrosLab.plotting.general) package.^15^

## Results

### Cell lines, tissue of origin and viral status

This section concerns the establishment of cell lines, confirmation of tissue of origin and HPV status. Short tandem repeat profiling of the patient's blood, the primary tumour and the developed cell line demonstrated a match for the majority of all 10 markers, confirming that the cell lines were indeed derived from the index patient and tumour (Appendix 1, available on *The Journal of Laryngology & Otology* website). In all cases, the short tandem repeat profiles matched exactly between the blood and primary tumour DNA; however, there were occasional differences at a single marker between the cell line and the parental tumour samples, likely because of minor drift as the cell line adapted to a plastic environment.

**Fig. 1.**
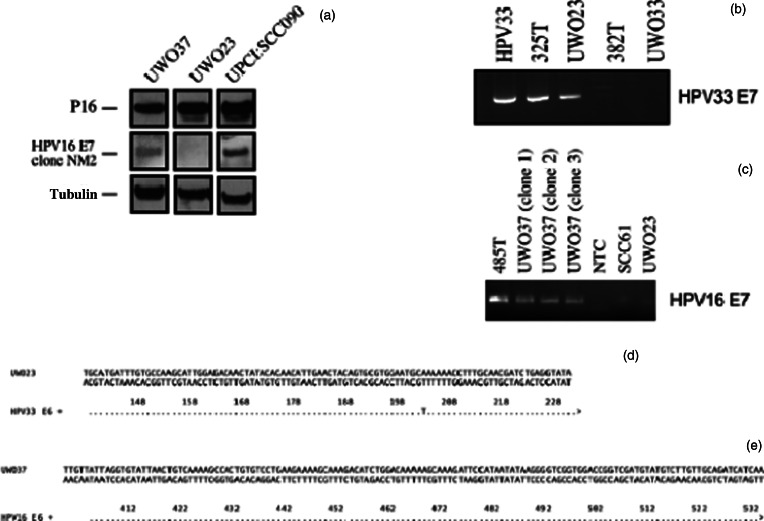
Determination of human papillomavirus (HPV) status in UWO23 and UWO37. (a) UWO23 and UWO37 were confirmed to express p16. UPCI:SCC090 was included as an HPV-positive control. The NM2 clone of the HPV16-specific antibody confirmed that UWO37 (and UPCI:SCC090) were HPV16-positive. The antibody did not cross-react with UWO23 (HPV33). Tubulin acted as a loading control. (b) Reverse transcription polymerase chain reaction confirmed that the parental tumour (325T) and derived cell line (UWO23) were positive for HPV33 E6 and E7 transcripts when compared to the positive control (HPV33). 382T (patient tumour) and UWO33 were a matched tumour/cell line pair not described in the manuscript, but used as an HPV-negative control for the specificity of the primers. (c) Reverse transcription polymerase chain reaction of the parental (pt 485) tumour and clones of the UWO37 cell line were positive HPV16 E7 transcripts. No HPV16 E7 transcripts were observed for UWO23 (HPV33 positive cell line), the negative control SCC61 or the ‘no template control’ (NTC). (d) UWO23 and (e) UWO37 alignment to E6 region of the appropriate HPV Genbank reference sequences. In (d), the UWO23 sequence is presented at the top and the HPV33 (HQ537706) sequence is aligned below. In (e), the UWO37 sequence is presented at the top and HPV16 (NC_001526) is aligned below. Dots indicate an exact nucleotide match at that position.

We tested the cell lines that we were able to establish for the presence of HPV DNA and transcripts. UWO23 was positive for HPV type 33 and UWO37 was positive for HPV type 16 on real-time polymerase chain reaction and Sanger sequencing. In addition, both lines expressed high levels of p16 on western blotting, as is commonly observed for HPV-positive head and neck cancers (Figure 1). UWO37 was positive for expression of HPV16 E7, although the signal was weak. In contrast, UWO23 E7 expression was not detected by this HPV16-specific antibody (Figure 1a).

**Fig. 2.**
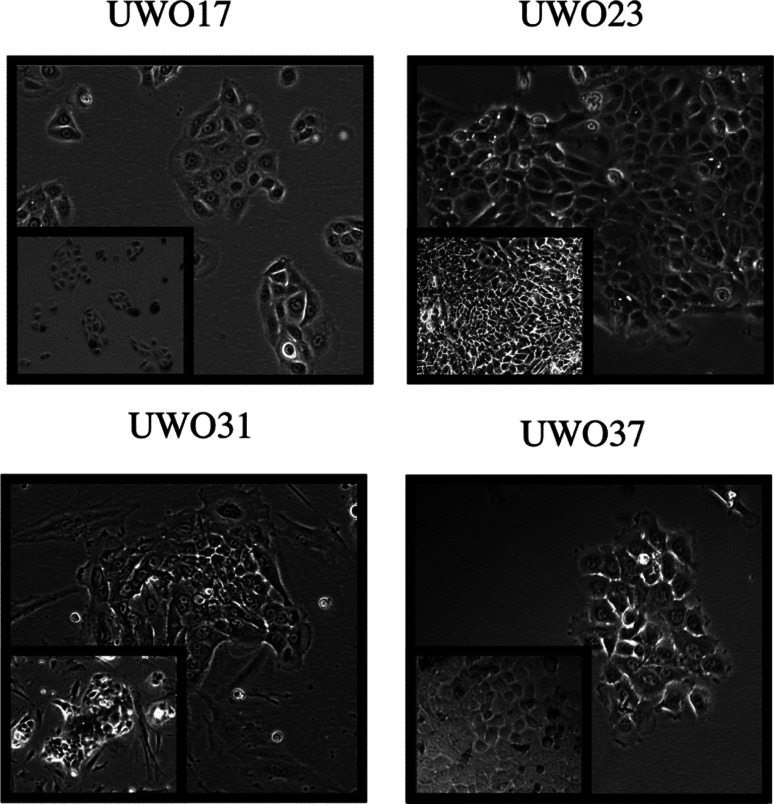
Cell morphology of the UWO primary cell lines. The four lines are shown at high magnification (10×) to illustrate the individual cell phenotype and colony organisation. The inset shows the cells at a higher density (lower magnification, 4×).

Although predominantly epithelial, cell line morphology varied (Figure 2). UWO17 cells grew in defined, loose colonies until they merged together into monolayers with even cell-to-cell spacing. They doubled in number every 40 ± 4 hours (mean **±** standard deviation) (Table 1). UWO23 cells exhibited an epithelial morphology and grew in tight monolayers. They doubled every 50 ± 6 hours. When passaged at low density, UWO31 cells grew in tight clusters that developed satellites of cells as the colonies spread. This line doubled approximately every 62 ± 6 hours. Stromal-like cells, in addition to epithelial cells, survived in these cultures. UWO37 grew as colonies of cells, and maintained this pattern even at high density. They doubled in number approximately every 41 ± 4 hours.

**Table 1.** Patient of origin details and cell line doubling time

Cell line	Site	Sex	Age at diagnosis (years)	HPV status	Tumour (T) stage	Nodal (N) stage	Treatment	Follow-up duration (years)	Last known status	Cell line doubling time (mean ± SD; hours)
UWO17	Oral tongue	F	58	Negative	2	3b	Surgery + chemoradiation	7	NED	40 ± 4
UWO23	Oral tongue	M	52	Positive	4	0	Surgery + chemoradiation	1.3	NED	50 ± 6
UWO31	Oral tongue	F	68	Negative	3	2a	Surgery + radiation	3.4	NED	62 ± 6
UWO37	Recurrent tonsil after chemoradiation	M	64	Positive	4	1	Surgery	1.1	DOD	41 ± 4

HPV = human papillomavirus; SD = standard deviation; F = female; M = male; NED = no evidence of disease; DOD = died of disease

### Matched reference DNA for cell line mutation calls

Matched reference DNA was essential for accurate cell line mutation calls. Whole exome sequencing was undertaken for the four tumours and their corresponding cell lines, and compared against the patient's reference leukocyte DNA (patient-matched). This was accomplished using the MuTect algorithm, where matched leukocyte DNA was employed as a reference versus using a panel of ‘normals’ generated from 438 Cancer Genome Atlas head and neck SCC samples (as described in the Methods section); this is in contrast to cell line mutation calls, which were performed against the matched leukocyte DNA. When we performed unmatched analysis, we observed an average of 314.5 true positive single nucleotide variants, 20.5 false negative single nucleotide variants and 2222.75 false positives relative to the gold standard paired analysis (Table 2). The false positive rate was 87.6 per cent and the false negative rate was 6.1 per cent.

**Table 2.** False positive and false negative mutational calls of cell lines identified using MuTect versus MuTect unpaired*

MuTect unpaired	MuTect paired	
	Positive	Negative
Positive	314.5	2222.75
Negative	20.5	N/A

Values reflect average numbers of mutational calls.

* With reference patient matched. N/A = not applicable

### Mutational landscape of early passage cell lines

**Fig. 3.**
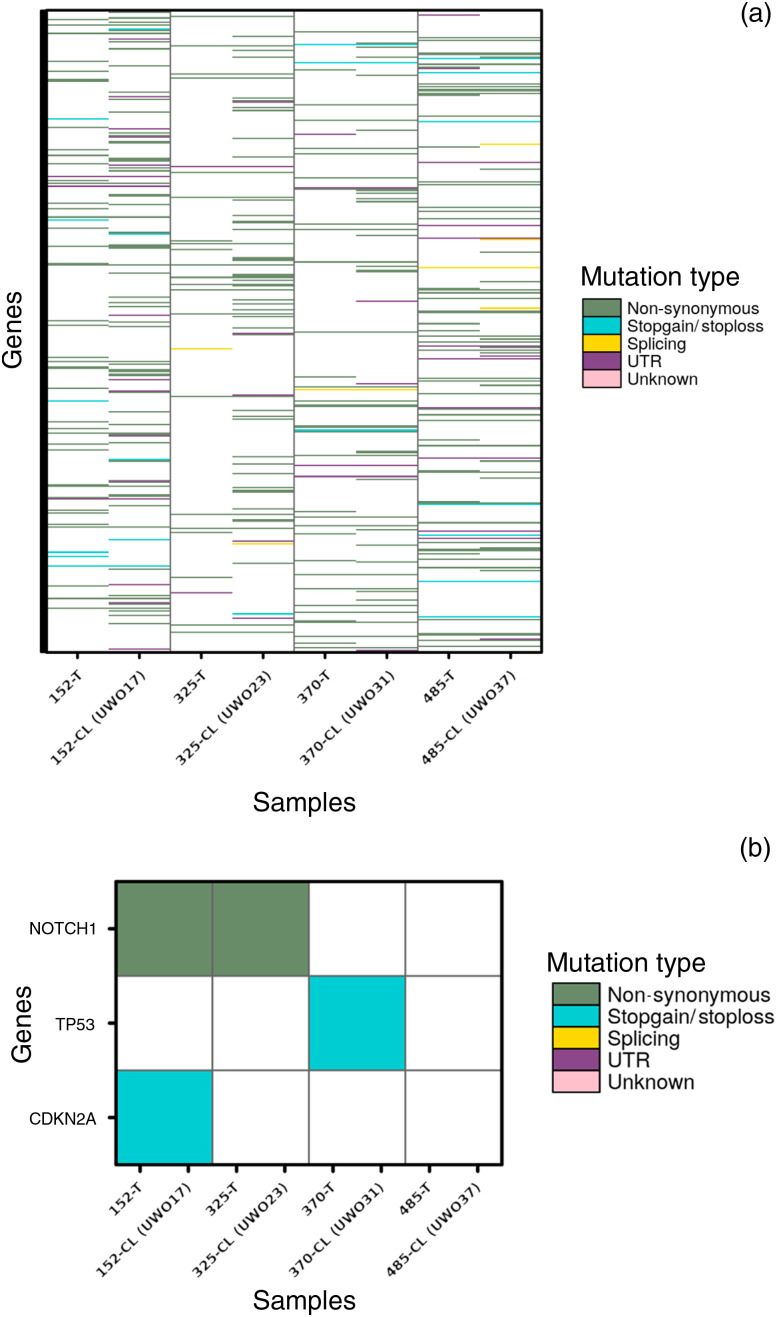
Mutational comparison of primary tumours and cell lines. (a) Heatmap of all single nucleotide variants identified by whole exome sequencing in four cell lines (CL) and their matched parental tumours (T). A full gene list is provided in Appendix 2 (available on *The Journal of Laryngology & Otology* website). (b) Single nucleotide variants identified in the top 20 most frequently mutated genes in the Cancer Genome Atlas head and neck squamous cell carcinoma study. Note that both human papillomavirus positive cell lines were TP53 wild-type as expected. UTR = untranslated region

**Fig. 4.**
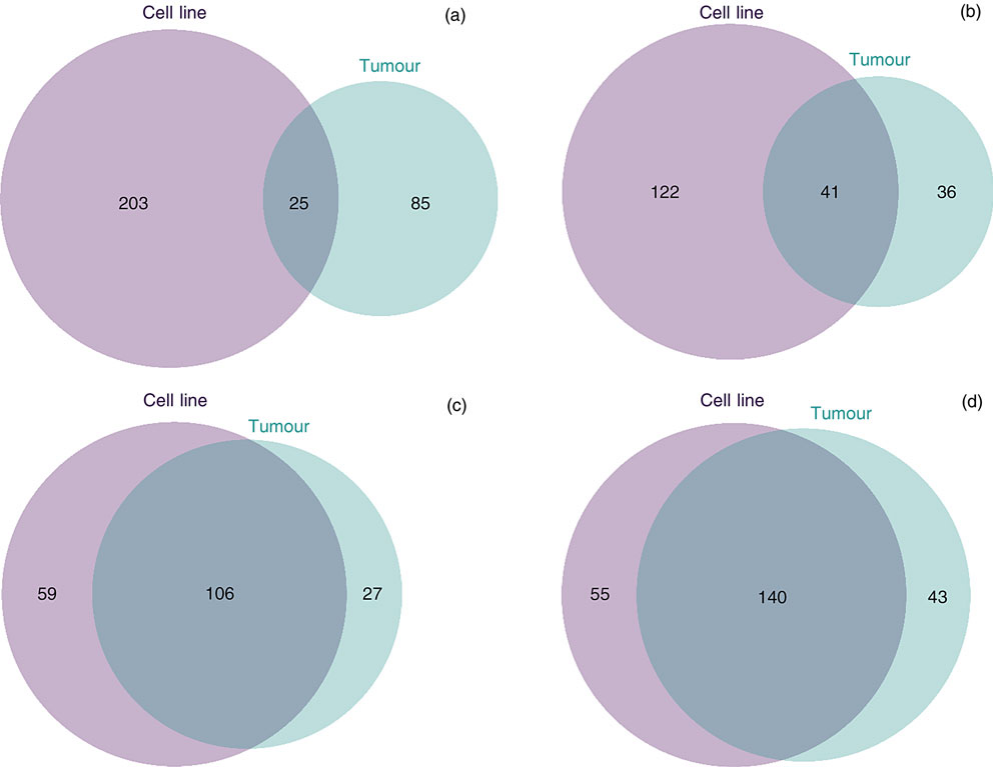
Overlap of the single nucleotide variants between tumour and cell line samples. (a) Patient 152, cell line UWO17. (b) Patient 325, cell line UWO23. (c) Patient 370, cell line UWO31. (d) Patient 485, cell line UWO37. Hypergeometric tests showed *p*-values of more than 0.05 for all samples, demonstrating that the paired cell lines and the parental tumours are genetically related.

Tumour and corresponding cell lines with reference patient matched DNA were analysed using SomaticSniper software (Figure 3a). For each corresponding tumour and cell line pair, the cell line exhibited more mutations than the contributing tumour, perhaps reflecting changes necessary for cells to adapt to culture conditions. When compared with the top 25 genes identified in the Cancer Genome Atlas marker paper,^16^ single nucleotide variants in *NOTCH1*, *TP53* and *CDKN2A* genes were identified (Figure 3b). A complete list of mutations in the cell lines and tumours is provided in Appendix 2, available on *The Journal of Laryngology & Otology* website. The hypergeometric distribution test was then applied to each tumour and cell line pair to assess whether two samples shared the same lineage based on mutation calls. For each of the paired samples, the *p*-value was not significant, suggesting that they did in fact share significantly more mutations than would occur by chance alone (Figure 4).

## Discussion

We have presented the establishment and molecular characterisation of four novel early passage head and neck SCC cell lines, including two HPV-positive lines. Importantly, we demonstrated that the reference patient-matched DNA from each patient is important to make accurate mutation calls. These patient-matched reference samples, as demonstrated in this study, allow accurate characterisation of cell lines. Furthermore, as observed in this study, bioinformatics pipelines are prone to errors with high false positive rates (87.6 per cent in our study). While cell lines and primary tumours shared significant numbers of specific mutations, demonstrating that they are related, both contained unique mutations, suggesting that genetic drift occurred in the cell lines as they adapted to culture conditions. It is also worth noting that the mutational calls between normal tissue and primary tumours that are shared are sufficient, but not necessary, for tumour transformation. These would be missed when using reference DNA.

There is an urgent need to identify novel chemicals with high activity in head and neck cancers in order to improve outcomes. Cancer cell lines are by far the most widely used model systems in cancer research, as they cost relatively little to maintain and can be easily manipulated in biological assays and feasibly screened with a large number of agents. Potentially, genomic characterisation of these cell lines could allow correlation of molecular features with drug response. Indeed, there have been two large-scale studies involving hundreds of genotyped cell lines of different tissue types,^6,7^ which have identified known, as well as novel, genetic markers of drug response.^6,7^ We specifically reanalysed the head and neck SCC data from both these studies and identified associations between PIK3CA mutations and response to a PI3K inhibitor; however, our analysis was limited by the small number of head and neck SCC lines and the limited number of drugs tested.^17^

In order to expand drug discovery and develop additional biomarkers, we previously screened 1433 agents in a panel of 26 head and neck SCC established cell lines, and identified drugs with preferential activity in HPV-positive and HPV-negative cell lines.^5,18^ However, our original intention was to correlate the cell line genomic profiles with drug response. We characterised our cell line panel with both OncoScan^™^ copy number arrays and whole exome sequencing, which our team has analysed extensively.^19–25^ While copy number arrays perform well without reference normal DNA,^22,24,26^ exome sequencing is prone to frequent false positives and negatives for both primary tumours and cell lines. In an effort to specifically address this, we developed a ‘reference-free somatic variant’ pipeline, which uses an extensive mutational ‘allowlist’ (known pathogenic variants) and ‘denylist’ (likely germline variants based on large population cohorts) as well as additional bioinformatics analysis.^27^ This pipeline is highly accurate in primary head and neck SCC and prostate tumours; however, despite exhaustive efforts, it does not perform well on cell lines.^27^ For this reason, we have started to generate a panel of both HPV-positive and HPV-negative cell lines from primary tumours, along with matched blood. To date, we have generated 17 such lines, and we have presented genomic analysis of the first 4 of these here.

An additional advantage of the cell lines described in this study is that they are early passage cell lines. Established cell lines have the distinct advantage of being broadly available to the cancer research community through established commercial culture collections. This offers the opportunity to reproduce data independently with the same reagents. Nevertheless, the correlation of established cell line drug responses with patient tumour responses appears to be modest,^28–30^ potentially because of additional molecular drift from the primary tumour profile through selection pressure in culture.^9,10^ Although beyond the scope of this manuscript with regard to comparison of early versus late passage cell lines, there is some evidence that early passage cell lines may represent superior models for drug screening.^28–30^ Thus, panels of genomically characterised early passage cell lines with patient-matched reference DNA, such as our novel University of Western Ontario panel, may be beneficial for the first phase of drug testing.

- Four early passage cell lines were established, and genomic characterisation was undertaken
- Two cell lines were human papillomavirus positive, confirmed by sequencing and P16 immunoblotting
- Short tandem repeat profiling confirmed that all four cell lines were established from their index tumours
- Whole exome sequencing revealed that matched normal reference DNA was critical for accurate mutational analysis: a high rate of false positive mutation calls (87.6 per cent) were excluded

The HPV-positive cell line models are of great interest to the head and neck research community in light of the epidemic of HPV-related oropharyngeal cancer.^12,31^ However, while numerous HPV-negative head and neck SCC cell lines exist, there is a paucity of HPV-positive lines.^32^ The ideal HPV-positive cell lines would be derived from non-smoking patients with a treatment-naïve oropharyngeal cancer; however, almost all lines, including our own, are derived from smokers, radiation failures (UWO37) or non-oropharyngeal primary sites (UWO23, oral cavity).^32^ Only recently has an ideal cell line been described.^33^ Further culture efforts are needed to generate additional models to facilitate drug discovery.

## Conclusion

We describe the establishment and characterisation of four head and neck SCC early passage cell lines with patient-matched reference DNA. These cell lines will act as a resource for drug discovery, as they can be genomically characterised and may be more accurate than late passage models. Development of additional early passage lines, particularly HPV-positive models, is needed.

## Supplementary material

For supplementary material accompanying this paper visit https://doi.org/10.1017/S0022215122000846. click here to view supplementary material

## References

[1] Weinstein JN. Drug discovery: cell lines battle cancer. Nature 2012;483:544–5 22460893 10.1038/483544a

[2] Simon R, Roychowdhury S. Implementing personalized cancer genomics in clinical trials. Nat Rev Drug Discov 2013;12:358–69 23629504 10.1038/nrd3979

[3] van Staveren WC, Solis DY, Hebrant A, Detours V, Dumont JE, Maenhaut C. Human cancer cell lines: experimental models for cancer cells in situ? *For cancer stem cells?* Biochim Biophys Acta 2009;1795:92–103 19167460 10.1016/j.bbcan.2008.12.004

[4] Bhandari V, Hoey C, Liu LY, Lalonde E, Ray J, Livingstone J Molecular landmarks of tumor hypoxia across cancer types. Nat Genet 2019;51:308–18 30643250 10.1038/s41588-018-0318-2

[5] Ghasemi F, Black M, Sun RX, Vizeacoumar F, Pinto N, Ruicci KM High-throughput testing in head and neck squamous cell carcinoma identifies agents with preferential activity in human papillomavirus-positive or negative cell lines. Oncotarget 2018;9:26064–71 29899842 10.18632/oncotarget.25436 PMC5995257

[6] Barretina J, Caponigro G, Stransky N, Venkatesan K, Margolin AA, Kim S The Cancer Cell Line Encyclopedia enables predictive modelling of anticancer drug sensitivity. Nature 2012;483:603–7 22460905 10.1038/nature11003 PMC3320027

[7] Garnett MJ, Edelman EJ, Heidorn SJ, Greenman CD, Dastur A, Lau KW Systematic identification of genomic markers of drug sensitivity in cancer cells. Nature 2012;483:570–5 22460902 10.1038/nature11005 PMC3349233

[8] Klijn C, Durinck S, Stawiski EW, Haverty PM, Jiang Z, Liu H A comprehensive transcriptional portrait of human cancer cell lines. Nat Biotechnol 2015;33:306–12 25485619 10.1038/nbt.3080

[9] O'Driscoll L, Gammell P, McKiernan E, Ryan E, Jeppesen PB, Rani S Phenotypic and global gene expression profile changes between low passage and high passage MIN-6 cells. J Endocrinol 2006;191:665–76 17170223 10.1677/joe.1.06894

[10] Wenger SL, Senft JR, Sargent LM, Bamezai R, Bairwa N, Grant SG. Comparison of established cell lines at different passages by karyotype and comparative genomic hybridization. Biosci Rep 2004;24:631–9 16158200 10.1007/s10540-005-2797-5

[11] Ruicci KM, Meens J, Sun RX, Rizzo G, Pinto N, Yoo J A controlled trial of HNSCC patient-derived xenografts reveals broad efficacy of PI3Kα inhibition in controlling tumor growth. Int J Cancer 2019;145:2100–6 30468243 10.1002/ijc.32009

[12] Nichols AC, Palma DA, Dhaliwal SS, Tan S, Theuer J, Chow W The epidemic of human papillomavirus and oropharyngeal cancer in a Canadian population. Curr Oncol 2013;20:212–19 23904762 10.3747/co.20.1375 PMC3728052

[13] Ruicci KM, Pinto N, Khan MI, Yoo J, Fung K, MacNeil D ERK-TSC2 signalling in constitutively-active HRAS mutant HNSCC cells promotes resistance to PI3K inhibition. Oral Oncol 2018;84:95–103 30115483 10.1016/j.oraloncology.2018.07.010

[14] Roth V. Doubling Time Computing, 2006. In: http://www.doubling-time.com/compute.php [10 November 2022]

[15] P'ng C, Green J, Chong LC, Waggott D, Prokopec SD, Shamsi M BPG: seamless, automated and interactive visualization of scientific data. BMC Bioinformatics 2019;20:42 30665349 10.1186/s12859-019-2610-2 PMC6341661

[16] Cancer Genome Atlas Network. Comprehensive genomic characterization of head and neck squamous cell carcinomas. Nature 2015;517:576–82 25631445 10.1038/nature14129 PMC4311405

[17] Nichols AC, Black M, Yoo J, Pinto N, Fernandes A, Haibe-Kains B Exploiting high-throughput cell line drug screening studies to identify candidate therapeutic agents in head and neck cancer. BMC Pharmacol Toxicol 2014;15:66 25428177 10.1186/2050-6511-15-66 PMC4258049

[18] Ghasemi F, Black M, Vizeacoumar F, Pinto N, Ruicci KM, Le C Repurposing Albendazole: new potential as a chemotherapeutic agent with preferential activity against HPV-negative head and neck squamous cell cancer. Oncotarget 2017;8:71512–19 29069723 10.18632/oncotarget.17292 PMC5641066

[19] Hua JT, Ahmed M, Guo H, Zhang Y, Chen S, Soares F Risk SNP-mediated promoter-enhancer switching drives prostate cancer through lncRNA PCAT19. Cell 2018;174:564–75.e18 30033362 10.1016/j.cell.2018.06.014

[20] Hopkins JF, Sabelnykova VY, Weischenfeldt J, Simon R, Aguiar JA, Alkallas R Mitochondrial mutations drive prostate cancer aggression. Nat Commun 2017;8:656 28939825 10.1038/s41467-017-00377-y PMC5610241

[21] Fraser M, Sabelnykova VY, Yamaguchi TN, Heisler LE, Livingstone J, Huang V Genomic hallmarks of localized, non-indolent prostate cancer. Nature 2017;541:359–64 28068672 10.1038/nature20788

[22] Boutros PC, Fraser M, Harding NJ, de Borja R, Trudel D, Lalonde E Spatial genomic heterogeneity within localized, multifocal prostate cancer. Nat Genet 2015;47:736–45 26005866 10.1038/ng.3315

[23] Weinreb I, Piscuoglio S, Martelotto LG, Waggott D, Ng CK, Perez-Ordonez B Hotspot activating PRKD1 somatic mutations in polymorphous low-grade adenocarcinomas of the salivary glands. Nat Genet 2014;46:1166–9 25240283 10.1038/ng.3096 PMC10208689

[24] Ewing AD, Houlahan KE, Hu Y, Ellrott K, Caloian C, Yamaguchi TN Combining tumor genome simulation with crowdsourcing to benchmark somatic single-nucleotide-variant detection. Nat Methods 2015;12:623–30 25984700 10.1038/nmeth.3407 PMC4856034

[25] Espiritu SMG, Liu LY, Rubanova Y, Bhandari V, Holgersen EM, Szyca LM The evolutionary landscape of localized prostate cancers drives clinical aggression. Cell 2018;173:1003–13.e15 29681457 10.1016/j.cell.2018.03.029

[26] Cooper CS, Eeles R, Wedge DC, Van Loo P, Gundem G, Alexandrov LB Analysis of the genetic phylogeny of multifocal prostate cancer identifies multiple independent clonal expansions in neoplastic and morphologically normal prostate tissue. Nat Genet 2015;47:367–72 25730763 10.1038/ng.3221 PMC4380509

[27] Sun R, Lalansingh C, Espiritu S, Yao C, Yamaguchi T, Prokopec S Accurate reference-free somatic variant-calling by integrating genomic, sequencing and population data. bioRxiv 2018. Epub 2018 August 2

[28] Domcke S, Sinha R, Levine DA, Sander C, Schultz N. Evaluating cell lines as tumour models by comparison of genomic profiles. Nat Commun 2013;4:2126 23839242 10.1038/ncomms3126 PMC3715866

[29] Baguley BC, Marshall ES. The use of human tumour cell lines in the discovery of new cancer chemotherapeutic drugs. Expert Opin Drug Discov 2008;3:153–61 23480219 10.1517/17460441.3.2.153

[30] Cree IA, Glaysher S, Harvey AL. Efficacy of anti-cancer agents in cell lines versus human primary tumour tissue. Curr Opin Pharmacol 2010;10:375–9 20570561 10.1016/j.coph.2010.05.001

[31] Chaturvedi AK, Engels EA, Pfeiffer RM, Hernandez BY, Xiao W, Kim E Human papillomavirus and rising oropharyngeal cancer incidence in the United States. J Clin Oncol 2011;29:4294–301 21969503 10.1200/JCO.2011.36.4596 PMC3221528

[32] Cheng H, Yang X, Si H, Saleh AD, Xiao W, Coupar J Genomic and transcriptomic characterization links cell lines with aggressive head and neck cancers. Cell Rep 2018;25:1332–45.e5 30380422 10.1016/j.celrep.2018.10.007 PMC6280671

[33] Forslund O, Sugiyama N, Wu C, Ravi N, Jin Y, Swoboda S A novel human in vitro papillomavirus type 16 positive tonsil cancer cell line with high sensitivity to radiation and cisplatin. BMC Cancer 2019;19:265 30909875 10.1186/s12885-019-5469-8 PMC6434888

