# Supplementary material for: Establishment of four head and neck squamous cell carcinoma cell lines: importance of reference DNA for accurate genomic characterisation
Source: J Laryngol Otol. 2022 Mar 23;137(3):301–7. doi: 10.1017/S0022215122000846 (PMC9975763; doi:10.1017/S0022215122000846)
Supplement: Supplementary file 1 [file S0022215122000846sup001.docx]

**Appendix 1.** Short tandem repeat profiles of primary cell lines and their parental tumour tissue (T) and control blood (B) from patients 152, 325, 370 and 485

| Parameter | AMEL | | CSF1PO | | D13S317 | | D16S539 | | D21S11 | | D5S818 | | D7S820 | | TH01 | | TPOX | | vWA | |
| --- | --- | --- | --- | --- | --- | --- | --- | --- | --- | --- | --- | --- | --- | --- | --- | --- | --- | --- | --- | --- |
| 152B | X | X | 10 | 12 | 12 | 13 | 11 | 12 | 29 | 32.2 | 12 | 13 | 9 | 12 | 7 | 8 | 10 | 11 | 17 | 18 |
| 152T | X | X | 10 | 12 | 12 | 13 | 11 | 12 | 29 | 32.2 | 12 | 13 | 9 | 12 | 7 | 8 | 10 | 11 | 17 | 18 |
| UWO17 | X | X | 10 | 12 | 12 | 13 | 11 | 12 | 29 | 32.2 | 12 | 13 | 12 | 12 | 7 | 8 | 10 | 10 | 17 | 18 |
|  |  |  |  |  |  |  |  |  |  |  |  |  |  |  |  |  |  |  |  |  |
| 325B | X | Y | 11 | 11 | 9 | 10 | 12 | 12 | 28 | 30 | 11 | 11 | 9 | 12 | 6 | 9 | 8 | 11 | 16 | 18 |
| 325T | X | Y | 11 | 11 | 9 | 10 | 12 | 12 | 28 | 30 | 11 | 11 | 9 | 12 | 6 | 9 | 8 | 11 | 16 | 18 |
| UWO23 | X | Y | 11 | 11 | 9 | 10 | 12 | 12 | 28 | 30 | 11 | 11 | 9 | 12 | 6 | 6 | 8 | 11 | 16 | 18 |
|  |  |  |  |  |  |  |  |  |  |  |  |  |  |  |  |  |  |  |  |  |
| 370B | X | X | 10 | 11 | 8 | 11 | 12 | 12 | 28 | 31.2 | 11 | 12 | 8 | 9 | 7 | 9 | 8 | 9 | 14 | 15 |
| 370T | X | X | 10 | 11 | 8 | 11 | 12 | 12 | 28 | 31.2 | 11 | 12 | 8 | 9 | 7 | 9 | 8 | 9 | 14 | 15 |
| UWO31 | X | X | 10 | 11 | 8 | 11 | 12 | 12 | 28 | 31.2 | 11 | 12 | 8 | 8 | 7 | 9 | 8 | 9 | 14 | 15 |
|  |  |  |  |  |  |  |  |  |  |  |  |  |  |  |  |  |  |  |  |  |
| 485B | X | Y | 9 | 11 | 12 | 12 | 9 | 12 | 27 | 34.2 | 12 | 13 | 10 | 12 | 7 | 9 | 8 | 10 | 16 | 17 |
| 485T | X | Y | 9 | 11 | 12 | 12 | 9 | 12 | 27 | 34.2 | 12 | 13 | 10 | 12 | 7 | 9 | 8 | 10 | 16 | 17 |
| UWO37 | X | Y | 9 | 11 | 11 | 12 | 9 | 12 | 27 | 34.2 | 12 | 13 | 10 | 10 | 7 | 9 | 8 | 10 | 16 | 17 |

**Appendix 2.** List of functional SNVs identified using SomaticSniper for parental tumours and corresponding cell lines

| SNV | 152T | UWO17 | 325T | UWO23 | 370T | UWO31 | 485T | UWO37 |
| --- | --- | --- | --- | --- | --- | --- | --- | --- |
| ABCB5 | N/A | N/A | N/A | 1 | N/A | N/A | N/A | N/A |
| ACOT8 | N/A | N/A | N/A | N/A | N/A | 4 | N/A | N/A |
| ACSL4 | N/A | 4 | N/A | N/A | N/A | N/A | N/A | N/A |
| ADAM10 | N/A | N/A | N/A | N/A | 1 | 1 | N/A | N/A |
| ADAMTS17 | N/A | N/A | N/A | N/A | N/A | N/A | 1 | 1 |
| ADAMTS19 | N/A | N/A | N/A | N/A | N/A | N/A | N/A | N/A |
| ADAMTSL2 | N/A | N/A | N/A | N/A | 1 | N/A | N/A | N/A |
| ADGRA2 | N/A | N/A | N/A | N/A | N/A | N/A | N/A | N/A |
| ADPRH | N/A | N/A | N/A | N/A | N/A | N/A | 1 | 1 |
| ADRA1A | N/A | N/A | N/A | N/A | N/A | N/A | N/A | 4 |
| AGAP2 | N/A | N/A | N/A | N/A | N/A | N/A | N/A | N/A |
| AGBL5 | N/A | N/A | N/A | N/A | 1 | 1 | N/A | N/A |
| AHNAK2 | N/A | N/A | N/A | N/A | N/A | N/A | 1 | 1 |
| AK7 | N/A | N/A | N/A | N/A | N/A | N/A | 1 | 1 |
| ALKAL2 | N/A | N/A | 1 | 1 | N/A | N/A | N/A | N/A |
| AMN1 | N/A | N/A | N/A | N/A | N/A | N/A | N/A | N/A |
| ANKLE1 | N/A | N/A | N/A | N/A | N/A | N/A | N/A | N/A |
| ANKRD18B | N/A | N/A | N/A | N/A | N/A | N/A | N/A | N/A |
| ANKRD27 | N/A | N/A | N/A | N/A | N/A | N/A | N/A | N/A |
| ANO9 | N/A | N/A | 1 | 1 | N/A | N/A | N/A | N/A |
| ANTXR1 | N/A | 1 | N/A | N/A | N/A | N/A | N/A | N/A |
| APBA2 | N/A | N/A | N/A | N/A | 1 | 1 | N/A | N/A |
| APOBEC3B | N/A | N/A | N/A | N/A | 1 | N/A | N/A | N/A |
| ARHGEF17 | N/A | N/A | N/A | N/A | N/A | N/A | 1 | 1 |
| ARHGEF9 | N/A | N/A | N/A | 4 | N/A | N/A | N/A | N/A |
| ARID4B | N/A | N/A | N/A | N/A | N/A | N/A | 2 | 2 |
| ARSD | N/A | 1 | N/A | N/A | N/A | N/A | N/A | N/A |
| ATP23 | N/A | N/A | N/A | 2 | N/A | N/A | N/A | N/A |
| ATP2B3 | N/A | N/A | N/A | N/A | 1 | 1 | N/A | N/A |
| AVL9 | N/A | 1 | N/A | N/A | N/A | N/A | N/A | N/A |
| B4GALNT4 | N/A | N/A | N/A | N/A | N/A | N/A | N/A | N/A |
| BAHD1 | 1 | 1 | N/A | N/A | N/A | N/A | N/A | N/A |
| BBS1 | N/A | N/A | N/A | N/A | N/A | N/A | N/A | N/A |
| BBX | N/A | N/A | N/A | N/A | 1 | 1 | N/A | N/A |
| BDP1 | N/A | 1 | N/A | N/A | N/A | N/A | N/A | N/A |
| BPIFA2 | N/A | 4 | N/A | N/A | N/A | N/A | N/A | N/A |
| BPIFB6 | N/A | N/A | N/A | N/A | N/A | N/A | N/A | N/A |
| BRD2 | N/A | N/A | N/A | N/A | N/A | 1 | N/A | N/A |
| BRIP1 | 1 | 1 | N/A | N/A | N/A | N/A | N/A | N/A |
| BRSK2 | N/A | N/A | N/A | N/A | N/A | N/A | N/A | N/A |
| BTAF1 | N/A | 1 | N/A | N/A | N/A | N/A | N/A | N/A |
| BTF3 | N/A | N/A | N/A | N/A | N/A | N/A | N/A | N/A |
| BTNL9 | N/A | N/A | 4 | N/A | N/A | N/A | N/A | N/A |
| C22orf34 | N/A | N/A | N/A | N/A | N/A | 1 | N/A | N/A |
| C2orf73 | N/A | N/A | N/A | N/A | N/A | N/A | N/A | N/A |
| C9orf172 | N/A | N/A | N/A | N/A | 1 | 1 | N/A | N/A |
| CA3 | N/A | N/A | N/A | N/A | N/A | N/A | N/A | N/A |
| CACNA1F | 1 | N/A | N/A | N/A | N/A | N/A | N/A | N/A |
| CAMKK2 | N/A | 4 | N/A | N/A | N/A | N/A | N/A | N/A |
| CAPSL | N/A | N/A | N/A | N/A | N/A | N/A | N/A | N/A |
| CCAR1 | N/A | N/A | N/A | N/A | N/A | N/A | 2 | 2 |
| CCBE1 | N/A | N/A | N/A | N/A | N/A | N/A | N/A | N/A |
| CCDC188 | N/A | N/A | N/A | N/A | N/A | N/A | N/A | N/A |
| CCL24 | N/A | N/A | 1 | N/A | N/A | N/A | N/A | N/A |
| CCT6B | N/A | N/A | N/A | N/A | N/A | N/A | N/A | N/A |
| CD248 | N/A | N/A | N/A | N/A | 1 | 1 | N/A | N/A |
| CD70 | N/A | N/A | N/A | N/A | N/A | N/A | N/A | N/A |
| CD96 | N/A | N/A | N/A | N/A | N/A | N/A | N/A | N/A |
| CDC42BPA | N/A | N/A | N/A | N/A | N/A | N/A | N/A | 1 |
| CDH18 | N/A | 1 | N/A | N/A | N/A | N/A | N/A | N/A |
| CDK13 | N/A | N/A | N/A | N/A | N/A | N/A | 1 | 1 |
| CDKN2A | 2 | 2 | N/A | N/A | N/A | N/A | N/A | N/A |
| CEACAM19 | N/A | N/A | N/A | N/A | N/A | N/A | N/A | N/A |
| CEP192 | N/A | N/A | N/A | 1 | N/A | N/A | N/A | N/A |
| CFAP54 | N/A | N/A | N/A | N/A | N/A | 1 | N/A | N/A |
| CIC | N/A | N/A | N/A | N/A | 1 | N/A | N/A | N/A |
| CILP2 | N/A | N/A | N/A | N/A | N/A | N/A | 1 | 1 |
| CKB | N/A | N/A | N/A | N/A | N/A | N/A | N/A | N/A |
| CLIP3 | 2 | N/A | N/A | N/A | N/A | N/A | N/A | N/A |
| CMYA5 | N/A | N/A | N/A | N/A | N/A | N/A | N/A | N/A |
| CNR2 | N/A | N/A | N/A | N/A | N/A | N/A | 1 | N/A |
| CNTN2 | 2 | N/A | N/A | N/A | N/A | N/A | N/A | N/A |
| CNTN5 | N/A | N/A | N/A | N/A | N/A | N/A | 1 | 1 |
| CNTNAP2 | N/A | N/A | N/A | N/A | N/A | N/A | 1 | 1 |
| CNTNAP4 | N/A | N/A | N/A | N/A | N/A | N/A | N/A | 1 |
| COG4 | N/A | N/A | N/A | N/A | N/A | N/A | N/A | N/A |
| COG7 | N/A | N/A | N/A | N/A | N/A | 1 | N/A | N/A |
| COL20A1 | N/A | N/A | N/A | 3 | N/A | N/A | N/A | N/A |
| COL6A5 | N/A | N/A | N/A | N/A | N/A | N/A | 1 | 1 |
| COX18 | N/A | N/A | N/A | 4 | N/A | N/A | N/A | N/A |
| CPNE4 | N/A | 2 | N/A | N/A | N/A | N/A | N/A | N/A |
| CPT1A | N/A | N/A | N/A | N/A | N/A | N/A | 4 | 4 |
| CPXCR1 | N/A | N/A | N/A | N/A | N/A | N/A | N/A | N/A |
| CREBBP | N/A | N/A | N/A | N/A | N/A | N/A | 2 | 2 |
| CROCC | N/A | N/A | N/A | N/A | 1 | N/A | N/A | N/A |
| CROCC2 | N/A | N/A | 1 | N/A | N/A | N/A | N/A | N/A |
| CRYBG1 | N/A | N/A | N/A | N/A | N/A | N/A | 4 | 4 |
| CSF2RA | N/A | N/A | N/A | N/A | N/A | 1 | N/A | N/A |
| CSMD1 | N/A | N/A | 1 | 1 | N/A | N/A | N/A | N/A |
| CSPG4 | 1 | 1 | N/A | N/A | N/A | N/A | N/A | N/A |
| CSRNP1 | N/A | N/A | N/A | N/A | 1 | 1 | N/A | N/A |
| CSTF1 | 1 | N/A | N/A | N/A | N/A | N/A | N/A | N/A |
| CTAGE1 | N/A | N/A | N/A | N/A | N/A | N/A | 1 | 1 |
| CTNNA2 | N/A | N/A | N/A | 1 | N/A | N/A | N/A | N/A |
| CTSA | N/A | N/A | N/A | 1 | N/A | N/A | N/A | N/A |
| CUBN | N/A | N/A | N/A | N/A | N/A | N/A | N/A | N/A |
| CXCL14 | N/A | N/A | N/A | N/A | 1 | 1 | N/A | N/A |
| CYP2A6 | N/A | N/A | N/A | N/A | N/A | N/A | N/A | N/A |
| DAAM1 | N/A | N/A | 1 | 1 | N/A | N/A | N/A | N/A |
| DBH | 1 | N/A | N/A | N/A | N/A | N/A | N/A | N/A |
| DBN1 | N/A | N/A | N/A | N/A | 1 | N/A | N/A | N/A |
| DBNL | 1 | N/A | N/A | N/A | N/A | N/A | N/A | N/A |
| DCHS2 | N/A | N/A | N/A | 1 | N/A | N/A | N/A | N/A |
| DCLRE1A | N/A | N/A | N/A | N/A | N/A | N/A | N/A | N/A |
| DDX1 | N/A | 1 | N/A | N/A | N/A | N/A | N/A | N/A |
| DDX3X | N/A | N/A | N/A | N/A | N/A | N/A | 2 | 2 |
| DDX52 | N/A | N/A | N/A | N/A | N/A | N/A | 1 | 1 |
| DIRC2 | N/A | N/A | N/A | N/A | N/A | N/A | 1 | N/A |
| DLC1 | N/A | N/A | N/A | N/A | N/A | N/A | N/A | N/A |
| DLX2 | 4 | 4 | N/A | N/A | N/A | N/A | N/A | N/A |
| DNAH5 | 1 | N/A | N/A | N/A | N/A | N/A | N/A | N/A |
| DNAH6 | N/A | 1 | N/A | N/A | N/A | N/A | N/A | N/A |
| DNAH7 | N/A | 1 | N/A | N/A | N/A | N/A | N/A | N/A |
| DNAJB3 | N/A | N/A | N/A | N/A | N/A | N/A | N/A | N/A |
| DOCK4 | N/A | N/A | N/A | 1 | N/A | N/A | N/A | N/A |
| DOCK9 | N/A | N/A | N/A | 1 | N/A | N/A | N/A | N/A |
| DPP7 | N/A | N/A | N/A | N/A | N/A | N/A | N/A | N/A |
| DPRX | N/A | N/A | N/A | 1 | N/A | N/A | N/A | N/A |
| DPYSL4 | 1 | 1 | N/A | N/A | N/A | N/A | N/A | N/A |
| DROSHA | 1 | N/A | N/A | N/A | N/A | N/A | N/A | N/A |
| DSP | N/A | N/A | N/A | N/A | N/A | N/A | N/A | N/A |
| DUSP16 | N/A | 1 | N/A | N/A | N/A | N/A | N/A | N/A |
| DUSP6 | N/A | 4 | N/A | N/A | N/A | N/A | N/A | N/A |
| EARS2 | N/A | N/A | N/A | N/A | N/A | 1 | N/A | N/A |
| EBI3 | N/A | N/A | N/A | N/A | N/A | N/A | N/A | 1 |
| EHHADH | N/A | N/A | N/A | N/A | 4 | 4 | N/A | N/A |
| EIF2B5 | N/A | N/A | N/A | N/A | N/A | N/A | N/A | N/A |
| EPB41L3 | N/A | N/A | N/A | 1 | N/A | N/A | N/A | N/A |
| EPYC | N/A | N/A | N/A | N/A | N/A | N/A | 1 | 1 |
| ERI1 | N/A | N/A | N/A | N/A | N/A | N/A | 1 | N/A |
| ESCO1 | N/A | N/A | N/A | N/A | N/A | N/A | N/A | N/A |
| ESRRG | N/A | N/A | N/A | N/A | N/A | N/A | N/A | N/A |
| FAM109B | N/A | N/A | N/A | N/A | N/A | N/A | N/A | N/A |
| FAM129A | N/A | N/A | N/A | N/A | 4 | 4 | N/A | N/A |
| FAM178B | N/A | N/A | N/A | 1 | N/A | N/A | N/A | N/A |
| FAM180A | N/A | N/A | N/A | N/A | N/A | N/A | N/A | N/A |
| FAM186A | N/A | 1 | N/A | N/A | N/A | N/A | N/A | 1 |
| FAM46B | N/A | 2 | N/A | N/A | N/A | N/A | N/A | N/A |
| FAM71C | N/A | N/A | N/A | N/A | N/A | N/A | 4 | 4 |
| FANCI | N/A | N/A | N/A | 1 | N/A | N/A | N/A | N/A |
| FAT4 | N/A | N/A | N/A | N/A | 1 | 1 | N/A | N/A |
| FBXO28 | N/A | N/A | N/A | N/A | N/A | N/A | N/A | N/A |
| FBXW2 | N/A | N/A | N/A | N/A | N/A | 1 | N/A | N/A |
| FCGBP | N/A | N/A | N/A | N/A | N/A | 1 | N/A | N/A |
| FGFR3 | 1 | N/A | N/A | N/A | N/A | N/A | N/A | N/A |
| FMN1 | N/A | N/A | N/A | N/A | N/A | N/A | N/A | N/A |
| FNBP4 | N/A | N/A | N/A | N/A | N/A | N/A | N/A | N/A |
| FNDC3B | N/A | N/A | N/A | N/A | N/A | N/A | 1 | 1 |
| FNDC8 | 1 | N/A | N/A | N/A | N/A | N/A | N/A | N/A |
| FNIP2 | N/A | 1 | N/A | N/A | N/A | N/A | N/A | N/A |
| FRMPD2 | N/A | N/A | N/A | N/A | N/A | N/A | N/A | N/A |
| FXYD4 | N/A | N/A | N/A | N/A | N/A | N/A | 1 | N/A |
| GATAD2A | N/A | N/A | N/A | N/A | N/A | N/A | N/A | N/A |
| GBP3 | N/A | N/A | N/A | N/A | N/A | N/A | N/A | N/A |
| GDI1 | N/A | 4 | N/A | N/A | N/A | N/A | N/A | N/A |
| GIPC1 | N/A | 1 | N/A | N/A | N/A | N/A | N/A | N/A |
| GLDC | 1 | 1 | N/A | N/A | N/A | N/A | N/A | N/A |
| GLYCTK | N/A | N/A | N/A | N/A | 1 | 1 | N/A | N/A |
| GMDS | N/A | N/A | N/A | N/A | 2 | 2 | N/A | N/A |
| GNB2 | 1 | N/A | N/A | N/A | N/A | N/A | N/A | N/A |
| GPC5 | N/A | N/A | N/A | N/A | 1 | 1 | N/A | N/A |
| GPC6 | N/A | N/A | N/A | N/A | 1 | 1 | N/A | N/A |
| GPR19 | N/A | 1 | N/A | N/A | N/A | N/A | N/A | N/A |
| GPR32 | N/A | N/A | N/A | N/A | N/A | N/A | N/A | N/A |
| GPT2 | 1 | N/A | N/A | N/A | N/A | N/A | N/A | N/A |
| GRIN2B | N/A | N/A | N/A | N/A | N/A | N/A | 1 | 1 |
| GRM5 | N/A | N/A | N/A | 1 | N/A | N/A | N/A | N/A |
| HABP2 | N/A | N/A | N/A | N/A | N/A | N/A | N/A | N/A |
| HAND2 | N/A | N/A | N/A | 1 | N/A | N/A | N/A | N/A |
| HECTD1 | N/A | N/A | N/A | N/A | N/A | N/A | 1 | 1 |
| HIRIP3 | N/A | N/A | N/A | N/A | N/A | N/A | N/A | N/A |
| HIST1H2BC | N/A | 1 | N/A | N/A | N/A | N/A | N/A | N/A |
| HLA-C | N/A | N/A | N/A | 1 | N/A | N/A | N/A | N/A |
| HM13 | N/A | N/A | N/A | N/A | N/A | N/A | 1 | 1 |
| HRASLS5 | N/A | N/A | N/A | N/A | N/A | N/A | 4 | 4 |
| HRNR | N/A | N/A | N/A | N/A | 1 | 1 | N/A | N/A |
| HSPD1 | N/A | N/A | N/A | N/A | 1 | 1 | N/A | N/A |
| HSPG2 | N/A | N/A | N/A | N/A | N/A | N/A | N/A | N/A |
| HTD2 | N/A | N/A | N/A | N/A | N/A | N/A | N/A | N/A |
| HUWE1 | 2 | N/A | N/A | N/A | 1 | 1 | N/A | N/A |
| HYKK | N/A | N/A | N/A | N/A | N/A | N/A | N/A | N/A |
| IFI30 | N/A | N/A | N/A | N/A | N/A | N/A | N/A | N/A |
| IFNLR1 | N/A | N/A | 1 | 1 | N/A | N/A | N/A | N/A |
| IFT74 | N/A | N/A | N/A | 4 | N/A | N/A | N/A | N/A |
| IGDCC3 | N/A | N/A | N/A | N/A | N/A | N/A | 1 | 1 |
| IGSF22 | N/A | 1 | N/A | N/A | N/A | N/A | N/A | N/A |
| IL31 | N/A | 4 | N/A | N/A | N/A | N/A | N/A | N/A |
| IMMT | N/A | N/A | N/A | N/A | 3 | 3 | N/A | N/A |
| INTS1 | 1 | N/A | N/A | N/A | N/A | N/A | N/A | N/A |
| INTS6L | N/A | N/A | N/A | N/A | 1 | 1 | N/A | N/A |
| IQCG | N/A | N/A | N/A | N/A | N/A | N/A | N/A | 1 |
| ITGA4 | N/A | N/A | N/A | N/A | N/A | 4 | N/A | N/A |
| JAK3 | N/A | N/A | N/A | N/A | N/A | N/A | N/A | N/A |
| JPH2 | N/A | N/A | N/A | N/A | N/A | N/A | 1 | 1 |
| JPT1 | N/A | 4 | N/A | N/A | N/A | N/A | N/A | N/A |
| KBTBD8 | N/A | N/A | N/A | N/A | N/A | N/A | 1 | 1 |
| KCNA10 | N/A | N/A | N/A | N/A | 1 | N/A | N/A | N/A |
| KCNA7 | N/A | 1 | N/A | N/A | N/A | N/A | N/A | N/A |
| KCNQ3 | N/A | 1 | N/A | N/A | N/A | N/A | N/A | N/A |
| KCNT2 | N/A | N/A | N/A | N/A | N/A | N/A | N/A | N/A |
| KCNU1 | N/A | 1 | N/A | N/A | N/A | N/A | N/A | N/A |
| KCTD9 | N/A | 1 | N/A | N/A | N/A | N/A | N/A | N/A |
| KIAA0586 | 1 | N/A | N/A | N/A | N/A | N/A | N/A | N/A |
| KIF25 | 1 | N/A | N/A | N/A | N/A | N/A | N/A | N/A |
| KIF2B | N/A | N/A | N/A | N/A | N/A | N/A | N/A | N/A |
| KIF3C | N/A | 1 | N/A | N/A | N/A | N/A | N/A | N/A |
| KIR3DL1,KIR3DS1 | N/A | N/A | N/A | N/A | N/A | N/A | N/A | N/A |
| KLHL21 | 1 | 1 | N/A | N/A | N/A | N/A | N/A | N/A |
| KLHL23 | N/A | N/A | N/A | N/A | N/A | N/A | N/A | N/A |
| KLHL32 | N/A | N/A | N/A | N/A | N/A | N/A | 4 | 4 |
| KLK11 | 1 | N/A | N/A | N/A | N/A | N/A | N/A | N/A |
| KMO | N/A | N/A | N/A | N/A | N/A | N/A | N/A | 4 |
| KRTAP1-3 | N/A | N/A | N/A | N/A | N/A | N/A | N/A | N/A |
| KRTAP9-2 | N/A | N/A | N/A | N/A | N/A | N/A | N/A | 1 |
| LAPTM4B | N/A | N/A | N/A | N/A | N/A | N/A | N/A | N/A |
| LEO1 | N/A | N/A | N/A | N/A | N/A | N/A | 1 | 1 |
| LINC00303 | N/A | N/A | 3 | N/A | N/A | N/A | N/A | N/A |
| LOC284898 | N/A | N/A | N/A | N/A | N/A | N/A | N/A | 1 |
| LPIN1 | N/A | N/A | N/A | N/A | N/A | N/A | 4 | 4 |
| LPIN2 | N/A | N/A | N/A | N/A | N/A | N/A | N/A | N/A |
| LRBA | N/A | N/A | N/A | N/A | N/A | N/A | N/A | N/A |
| LRP1B | N/A | N/A | N/A | N/A | N/A | N/A | 1 | 1 |
| LRRC49 | N/A | N/A | N/A | N/A | N/A | N/A | N/A | N/A |
| LRRC7 | N/A | N/A | N/A | N/A | N/A | N/A | N/A | 1 |
| LRRC75B | N/A | N/A | N/A | N/A | N/A | N/A | N/A | N/A |
| LRRFIP1 | N/A | N/A | N/A | N/A | N/A | N/A | 1 | 1 |
| LTN1 | N/A | N/A | N/A | 1 | N/A | N/A | N/A | N/A |
| LXN | N/A | N/A | N/A | 4 | N/A | N/A | N/A | N/A |
| MADD | N/A | N/A | N/A | N/A | 1 | 1 | N/A | N/A |
| MAGEC1 | N/A | N/A | N/A | N/A | N/A | N/A | 1 | N/A |
| MAML2 | N/A | N/A | N/A | N/A | N/A | N/A | N/A | N/A |
| MAP1B | N/A | N/A | N/A | 1 | N/A | N/A | N/A | N/A |
| MAP2 | N/A | 1 | N/A | N/A | N/A | N/A | N/A | N/A |
| MAP3K6 | 1 | N/A | N/A | N/A | N/A | N/A | N/A | N/A |
| MAPK15 | N/A | N/A | N/A | N/A | N/A | N/A | 1 | N/A |
| MAST2 | N/A | 1 | N/A | N/A | N/A | N/A | N/A | N/A |
| MB21D2 | 1 | N/A | N/A | N/A | N/A | N/A | N/A | N/A |
| MEF2C | N/A | N/A | N/A | N/A | N/A | N/A | N/A | N/A |
| MEGF10 | N/A | N/A | N/A | N/A | N/A | N/A | N/A | N/A |
| MEGF6 | N/A | N/A | N/A | 1 | N/A | N/A | N/A | N/A |
| MEIS3 | N/A | 4 | N/A | N/A | N/A | N/A | N/A | N/A |
| MGAM2 | N/A | N/A | 1 | 1 | N/A | N/A | N/A | N/A |
| MINK1 | N/A | N/A | N/A | N/A | N/A | N/A | 1 | 1 |
| MMAA | N/A | N/A | N/A | N/A | N/A | N/A | 1 | 1 |
| MMEL1 | N/A | N/A | N/A | 1 | N/A | N/A | N/A | N/A |
| MMS22L | N/A | N/A | N/A | N/A | N/A | N/A | N/A | 3 |
| MPDZ | N/A | 1 | N/A | N/A | N/A | N/A | N/A | N/A |
| MPP3 | N/A | N/A | N/A | N/A | N/A | N/A | N/A | N/A |
| MRC1 | N/A | N/A | N/A | 1 | N/A | N/A | N/A | N/A |
| MROH2B | N/A | 1 | N/A | N/A | N/A | N/A | N/A | N/A |
| MRPL2 | N/A | N/A | N/A | N/A | N/A | 4 | N/A | N/A |
| MRPS2 | N/A | N/A | N/A | 1 | N/A | N/A | N/A | N/A |
| MSX1 | N/A | N/A | N/A | N/A | N/A | N/A | 1 | 1 |
| MTM1 | N/A | 1 | N/A | N/A | N/A | N/A | N/A | N/A |
| MTNR1B | N/A | N/A | N/A | N/A | N/A | N/A | N/A | N/A |
| MTUS2 | N/A | N/A | N/A | N/A | N/A | N/A | N/A | N/A |
| MUC12 | N/A | N/A | N/A | N/A | N/A | N/A | 1 | 1 |
| MUC2 | N/A | N/A | N/A | N/A | N/A | N/A | N/A | N/A |
| MUC4 | N/A | N/A | 1 | 1 | N/A | N/A | 1 | 1 |
| MUC4;MUC4(NM_001322468:exon15:c.9770-1G>T) | N/A | N/A | N/A | N/A | N/A | N/A | 1 | N/A |
| MUC6 | N/A | N/A | N/A | N/A | N/A | N/A | N/A | N/A |
| MYLK | N/A | N/A | N/A | 1 | N/A | N/A | N/A | N/A |
| MYLPF | N/A | N/A | 1 | 1 | N/A | N/A | N/A | N/A |
| MYO15B | N/A | N/A | N/A | N/A | N/A | N/A | N/A | N/A |
| MYO1B | N/A | N/A | N/A | N/A | N/A | N/A | N/A | 1 |
| MYO7A | N/A | N/A | N/A | 1 | N/A | N/A | N/A | N/A |
| NAA10 | N/A | N/A | N/A | N/A | N/A | N/A | N/A | N/A |
| NABP2 | N/A | N/A | 1 | 1 | N/A | N/A | N/A | N/A |
| NAP1L4 | N/A | N/A | N/A | 1 | N/A | N/A | N/A | N/A |
| NAV1 | N/A | N/A | N/A | 1 | N/A | N/A | N/A | N/A |
| NCKAP1 | N/A | 1 | N/A | N/A | N/A | N/A | N/A | N/A |
| NCOA3 | N/A | N/A | N/A | N/A | N/A | 1 | N/A | N/A |
| NDST4 | N/A | N/A | N/A | N/A | N/A | 1 | N/A | N/A |
| NEB | N/A | N/A | N/A | N/A | N/A | N/A | N/A | N/A |
| NFASC | N/A | N/A | N/A | N/A | N/A | N/A | 3 | 3 |
| NONO | N/A | N/A | N/A | N/A | N/A | 1 | N/A | N/A |
| NOTCH1 | 1 | 1 | 1 | 1 | N/A | N/A | N/A | N/A |
| NPAS1 | 1 | N/A | N/A | N/A | N/A | N/A | N/A | N/A |
| NRAP | N/A | N/A | N/A | N/A | 1 | 1 | N/A | N/A |
| NRDC | N/A | N/A | N/A | 1 | N/A | N/A | N/A | N/A |
| NSD3 | N/A | N/A | N/A | 1 | N/A | N/A | N/A | N/A |
| NWD2 | N/A | N/A | N/A | 1 | N/A | N/A | N/A | N/A |
| NYX | N/A | N/A | N/A | N/A | N/A | N/A | N/A | N/A |
| OBSCN | N/A | N/A | N/A | N/A | N/A | N/A | N/A | N/A |
| OR2A7 | N/A | N/A | N/A | N/A | N/A | N/A | N/A | N/A |
| OR2L8 | N/A | N/A | N/A | N/A | N/A | N/A | N/A | 1 |
| OR2T8 | N/A | N/A | N/A | N/A | N/A | N/A | N/A | N/A |
| OR4N2 | N/A | N/A | 1 | N/A | N/A | N/A | N/A | N/A |
| OR5K3 | N/A | 1 | N/A | N/A | N/A | N/A | N/A | N/A |
| OSBPL1A | 1 | 1 | N/A | N/A | N/A | N/A | N/A | N/A |
| OTOP3 | N/A | 1 | N/A | N/A | N/A | N/A | N/A | N/A |
| OXCT1 | N/A | N/A | 1 | 1 | N/A | N/A | N/A | N/A |
| PACRG | N/A | N/A | N/A | N/A | N/A | N/A | N/A | N/A |
| PARP4 | N/A | N/A | 1 | N/A | N/A | N/A | N/A | N/A |
| PCDH11Y | N/A | N/A | N/A | N/A | N/A | N/A | N/A | 3 |
| PCDHA11 | N/A | N/A | N/A | N/A | N/A | N/A | 4 | 4 |
| PCDHGA9 | N/A | N/A | N/A | N/A | N/A | N/A | N/A | N/A |
| PCLO | N/A | N/A | N/A | N/A | N/A | 1 | N/A | N/A |
| PCSK1 | N/A | 2 | N/A | N/A | N/A | N/A | N/A | N/A |
| PCSK5 | N/A | 1 | N/A | N/A | N/A | N/A | N/A | N/A |
| PDCD10 | N/A | N/A | N/A | N/A | N/A | N/A | N/A | N/A |
| PDE3A | N/A | N/A | N/A | 1 | 1 | 1 | N/A | N/A |
| PDE4D | 1 | N/A | N/A | N/A | N/A | N/A | N/A | N/A |
| PERM1 | N/A | N/A | N/A | N/A | N/A | N/A | N/A | N/A |
| PFKFB4 | N/A | N/A | N/A | N/A | N/A | N/A | 4 | 4 |
| PHKA2 | N/A | N/A | N/A | N/A | 1 | 1 | N/A | N/A |
| PIGW | N/A | N/A | N/A | 1 | N/A | N/A | N/A | N/A |
| PKDREJ | N/A | N/A | N/A | 1 | N/A | N/A | N/A | N/A |
| PKP3 | 2 | N/A | N/A | N/A | N/A | N/A | N/A | N/A |
| PLD1 | N/A | N/A | N/A | N/A | N/A | N/A | 1 | 1 |
| PLEKHA3 | 1 | 1 | N/A | N/A | N/A | N/A | N/A | N/A |
| PLEKHG3 | N/A | N/A | N/A | 1 | N/A | N/A | N/A | N/A |
| PLIN4 | N/A | N/A | N/A | N/A | N/A | N/A | N/A | N/A |
| PLK3 | N/A | N/A | N/A | N/A | N/A | N/A | N/A | N/A |
| PLOD1 | N/A | N/A | N/A | N/A | N/A | N/A | 1 | 1 |
| PLXDC1 | N/A | N/A | N/A | N/A | N/A | N/A | N/A | N/A |
| PLXNB2 | 1 | N/A | N/A | N/A | N/A | N/A | N/A | N/A |
| PNLDC1 | N/A | N/A | N/A | N/A | N/A | N/A | 1 | 1 |
| PODXL | N/A | N/A | N/A | N/A | N/A | N/A | N/A | N/A |
| POLR2G | N/A | 1 | N/A | N/A | N/A | N/A | N/A | N/A |
| PON1 | 1 | N/A | N/A | N/A | N/A | N/A | N/A | N/A |
| PRR32 | N/A | N/A | N/A | N/A | N/A | 1 | N/A | N/A |
| PTGFR | N/A | N/A | N/A | 1 | N/A | N/A | N/A | N/A |
| PTPRF | N/A | N/A | N/A | N/A | N/A | 1 | N/A | N/A |
| PTPRO | N/A | N/A | 1 | 1 | N/A | N/A | N/A | N/A |
| PTPRU | N/A | 1 | N/A | N/A | N/A | N/A | N/A | N/A |
| RAB26 | N/A | 1 | N/A | N/A | N/A | N/A | N/A | N/A |
| RABL6 | N/A | N/A | N/A | N/A | N/A | 1 | N/A | N/A |
| RAD54L2 | N/A | N/A | N/A | N/A | N/A | N/A | N/A | N/A |
| RAG1 | N/A | N/A | N/A | N/A | N/A | 1 | N/A | N/A |
| RALYL | N/A | N/A | N/A | N/A | 1 | 1 | N/A | N/A |
| RANBP1 | N/A | N/A | N/A | N/A | 4 | 4 | N/A | N/A |
| RASA1 | 4 | 4 | N/A | N/A | N/A | N/A | N/A | N/A |
| RASGRP3 | N/A | N/A | N/A | N/A | N/A | N/A | 1 | 1 |
| RAVER2 | 1 | 1 | N/A | N/A | N/A | N/A | N/A | N/A |
| RBM17 | N/A | N/A | N/A | N/A | N/A | N/A | 1 | 1 |
| REM1 | 1 | N/A | N/A | N/A | N/A | N/A | N/A | N/A |
| REPIN1 | N/A | N/A | N/A | N/A | N/A | N/A | N/A | N/A |
| REV1 | N/A | N/A | N/A | N/A | 1 | 1 | N/A | N/A |
| RFC2 | 4 | 4 | N/A | N/A | N/A | N/A | N/A | N/A |
| RFPL1 | N/A | N/A | N/A | N/A | N/A | N/A | N/A | N/A |
| RGL3 | N/A | N/A | N/A | N/A | N/A | N/A | N/A | N/A |
| RGS22 | N/A | N/A | 1 | 1 | N/A | N/A | N/A | N/A |
| RHBG | N/A | 1 | N/A | N/A | N/A | N/A | N/A | N/A |
| RHOBTB1 | N/A | N/A | N/A | N/A | N/A | N/A | N/A | 1 |
| RIBC2 | N/A | 1 | N/A | N/A | N/A | N/A | N/A | N/A |
| RMI1 | N/A | N/A | 4 | 4 | N/A | N/A | N/A | N/A |
| RNASE13 | N/A | 4 | N/A | N/A | N/A | N/A | N/A | N/A |
| RNF111 | 1 | N/A | N/A | N/A | N/A | N/A | N/A | N/A |
| RNF130 | N/A | N/A | N/A | N/A | N/A | N/A | 4 | 4 |
| RNF24 | N/A | 1 | N/A | N/A | N/A | N/A | N/A | N/A |
| RPL10 | N/A | 1 | N/A | N/A | N/A | N/A | N/A | N/A |
| RTL5 | N/A | 1 | N/A | N/A | N/A | N/A | N/A | N/A |
| RUNX1T1 | N/A | N/A | N/A | 1 | N/A | N/A | N/A | N/A |
| RWDD2A | 1 | N/A | N/A | N/A | N/A | N/A | N/A | N/A |
| RYR1 | N/A | N/A | N/A | N/A | 1 | 1 | N/A | N/A |
| SALL2 | N/A | N/A | N/A | N/A | N/A | N/A | N/A | N/A |
| SALL3 | N/A | N/A | N/A | N/A | N/A | N/A | N/A | N/A |
| SAMD9L | 1 | N/A | N/A | N/A | N/A | N/A | N/A | N/A |
| SCAPER | N/A | N/A | N/A | N/A | 1 | 1 | N/A | N/A |
| SCARA5 | N/A | N/A | N/A | N/A | N/A | N/A | 1 | N/A |
| SCLT1 | N/A | N/A | N/A | N/A | N/A | N/A | N/A | N/A |
| SCN11A | N/A | N/A | N/A | N/A | N/A | N/A | N/A | 3 |
| SDHAF2 | N/A | 1 | N/A | N/A | N/A | N/A | N/A | N/A |
| SEMA4C | N/A | 1 | N/A | N/A | N/A | N/A | N/A | N/A |
| SEPHS2 | N/A | N/A | N/A | N/A | N/A | N/A | N/A | N/A |
| SETD5 | N/A | N/A | N/A | 1 | N/A | N/A | N/A | N/A |
| SF3B3 | N/A | 4 | N/A | N/A | N/A | N/A | N/A | N/A |
| SH2D3C | N/A | 1 | N/A | N/A | N/A | N/A | N/A | N/A |
| SH3D21 | N/A | N/A | N/A | N/A | 4 | N/A | N/A | N/A |
| SIGLEC1 | N/A | 1 | N/A | N/A | N/A | N/A | N/A | N/A |
| SLC10A1 | N/A | N/A | N/A | N/A | N/A | N/A | N/A | N/A |
| SLC22A4 | N/A | N/A | N/A | N/A | N/A | 1 | N/A | N/A |
| SLC25A2 | N/A | 4 | N/A | N/A | N/A | N/A | N/A | N/A |
| SLC25A30 | 1 | N/A | N/A | N/A | N/A | N/A | N/A | N/A |
| SLC2A9 | N/A | N/A | N/A | N/A | N/A | N/A | N/A | N/A |
| SLC33A1 | N/A | N/A | N/A | N/A | N/A | N/A | N/A | N/A |
| SLC4A4 | N/A | N/A | N/A | N/A | N/A | N/A | N/A | N/A |
| SLC6A16 | N/A | N/A | N/A | N/A | N/A | N/A | 2 | 2 |
| SLC6A5 | N/A | N/A | N/A | N/A | 1 | 1 | N/A | N/A |
| SLC9A1 | 2 | N/A | N/A | N/A | N/A | N/A | N/A | N/A |
| SLCO1B3 | N/A | N/A | N/A | N/A | N/A | N/A | N/A | N/A |
| SLIT2 | N/A | N/A | N/A | N/A | N/A | N/A | 1 | 1 |
| SMPD3 | N/A | N/A | N/A | N/A | N/A | N/A | N/A | N/A |
| SNUPN | N/A | N/A | N/A | N/A | N/A | N/A | N/A | N/A |
| SOD1 | N/A | 1 | N/A | N/A | N/A | N/A | N/A | N/A |
| SP8 | N/A | N/A | N/A | 1 | N/A | N/A | N/A | N/A |
| SPACA1 | N/A | N/A | N/A | N/A | N/A | N/A | N/A | N/A |
| SPG11 | N/A | N/A | N/A | 1 | N/A | N/A | N/A | N/A |
| SPON2 | N/A | N/A | N/A | N/A | N/A | N/A | N/A | N/A |
| SPTA1 | N/A | N/A | N/A | N/A | 1 | 1 | N/A | N/A |
| SPTAN1 | N/A | N/A | N/A | N/A | N/A | N/A | N/A | N/A |
| SPX | N/A | N/A | N/A | 4 | N/A | N/A | N/A | N/A |
| SSPO | N/A | N/A | N/A | 1 | N/A | N/A | N/A | N/A |
| STK33 | N/A | 1 | N/A | N/A | N/A | N/A | N/A | N/A |
| STT3A | N/A | N/A | N/A | 1 | N/A | N/A | N/A | N/A |
| SUPT5H | N/A | 4 | N/A | N/A | N/A | N/A | N/A | N/A |
| SUSD2 | N/A | N/A | N/A | N/A | N/A | N/A | 1 | N/A |
| SWI5 | N/A | N/A | N/A | N/A | N/A | N/A | 1 | 1 |
| TAAR5 | N/A | 1 | N/A | N/A | N/A | N/A | N/A | N/A |
| TAB2 | N/A | N/A | N/A | N/A | N/A | N/A | 1 | 1 |
| TAS1R1 | N/A | N/A | N/A | N/A | N/A | N/A | 1 | 1 |
| TAS2R43 | N/A | N/A | N/A | N/A | N/A | N/A | N/A | N/A |
| TBC1D30 | N/A | N/A | N/A | N/A | N/A | N/A | 1 | 1 |
| TCEA3 | N/A | N/A | N/A | N/A | N/A | N/A | N/A | N/A |
| TCP11 | N/A | N/A | N/A | N/A | N/A | N/A | 1 | 1 |
| TEP1 | N/A | N/A | N/A | N/A | N/A | N/A | N/A | N/A |
| TEPSIN | 1 | N/A | N/A | N/A | N/A | N/A | N/A | N/A |
| TESK1 | 1 | N/A | N/A | N/A | N/A | N/A | N/A | N/A |
| TEX45 | N/A | N/A | 1 | 1 | N/A | N/A | N/A | N/A |
| TFB1M | N/A | N/A | N/A | N/A | N/A | N/A | N/A | N/A |
| TFEB | N/A | N/A | N/A | N/A | N/A | 1 | N/A | N/A |
| TLR5 | N/A | N/A | 1 | 1 | N/A | N/A | N/A | N/A |
| TMEM159 | N/A | N/A | N/A | N/A | N/A | N/A | 2 | 2 |
| TMEM181 | 1 | N/A | N/A | N/A | N/A | N/A | N/A | N/A |
| TMEM247 | N/A | N/A | N/A | N/A | 1 | N/A | N/A | N/A |
| TMEM39B | N/A | N/A | N/A | N/A | N/A | N/A | 4 | N/A |
| TMPRSS9 | N/A | N/A | N/A | N/A | N/A | N/A | 1 | N/A |
| TNIK | N/A | 1 | N/A | N/A | N/A | N/A | N/A | N/A |
| TOP3A | N/A | N/A | N/A | N/A | N/A | N/A | 1 | 1 |
| TP53 | N/A | N/A | N/A | N/A | 2 | 2 | N/A | N/A |
| TRAPPC10 | 1 | N/A | N/A | N/A | N/A | N/A | N/A | N/A |
| TRAPPC9 | N/A | N/A | N/A | N/A | N/A | N/A | N/A | N/A |
| TRIP12 | N/A | N/A | N/A | N/A | N/A | N/A | 2 | 2 |
| TTN | N/A | N/A | N/A | 1 | N/A | 1 | N/A | 1 |
| TXLNG | N/A | N/A | N/A | N/A | N/A | N/A | N/A | N/A |
| UBA7 | N/A | N/A | N/A | N/A | N/A | N/A | 1 | 1 |
| UBE2N | N/A | N/A | N/A | N/A | N/A | N/A | 1 | 1 |
| UBXN4 | N/A | N/A | N/A | N/A | N/A | N/A | N/A | N/A |
| UNC13C | N/A | N/A | N/A | 1 | N/A | N/A | N/A | N/A |
| UNC80 | N/A | 1 | N/A | N/A | N/A | N/A | N/A | N/A |
| USP1 | N/A | N/A | N/A | N/A | N/A | 1 | N/A | N/A |
| USP38 | N/A | N/A | 1 | 1 | N/A | N/A | N/A | N/A |
| UTP3 | N/A | N/A | N/A | N/A | 2 | 2 | N/A | N/A |
| VAV1 | N/A | N/A | N/A | N/A | N/A | 1 | N/A | N/A |
| VDAC2 | N/A | 1 | N/A | N/A | N/A | N/A | N/A | N/A |
| VSX1 | N/A | N/A | N/A | N/A | N/A | N/A | 1 | 1 |
| VWA5A | N/A | 4 | N/A | N/A | N/A | N/A | N/A | N/A |
| VWF | N/A | N/A | N/A | N/A | N/A | N/A | 1 | 1 |
| WDR72 | N/A | N/A | N/A | 1 | N/A | N/A | N/A | N/A |
| WIPF3 | N/A | N/A | N/A | N/A | N/A | N/A | N/A | N/A |
| WNK4 | 1 | 1 | N/A | N/A | N/A | N/A | N/A | N/A |
| XIRP1 | N/A | N/A | N/A | N/A | 1 | 1 | N/A | N/A |
| YTHDF1 | N/A | 1 | N/A | N/A | N/A | N/A | N/A | N/A |
| ZC3H18 | N/A | 2 | N/A | N/A | N/A | N/A | N/A | N/A |
| ZDHHC11 | N/A | N/A | N/A | N/A | N/A | N/A | N/A | N/A |
| ZDHHC8 | N/A | N/A | N/A | N/A | N/A | N/A | N/A | N/A |
| ZEB1 | 1 | 1 | N/A | N/A | N/A | N/A | N/A | N/A |
| ZER1 | N/A | N/A | N/A | N/A | N/A | N/A | N/A | N/A |
| ZFHX4 | N/A | 1 | N/A | N/A | N/A | N/A | N/A | N/A |
| ZFP92 | N/A | N/A | N/A | N/A | N/A | N/A | N/A | N/A |
| ZNF182 | 1 | N/A | N/A | N/A | N/A | N/A | N/A | N/A |
| ZNF398 | N/A | 1 | N/A | N/A | N/A | N/A | N/A | N/A |
| ZNF536 | N/A | N/A | 1 | 1 | N/A | N/A | N/A | N/A |
| ZNF563 | N/A | N/A | N/A | N/A | N/A | N/A | 4 | N/A |
| ZNF618 | N/A | N/A | N/A | N/A | N/A | N/A | N/A | N/A |
| ZNF736 | N/A | 1 | N/A | N/A | N/A | N/A | N/A | N/A |
| ZNF830 | N/A | 1 | N/A | N/A | N/A | N/A | N/A | N/A |

SNV = single nucleotide variant; N/A = not applicable
